# Late Recognition of Suspected Ehlers‐Danlos Syndrome After Recurrent Small‐Bowel Perforations: A Case Report

**DOI:** 10.1002/jgf2.70183

**Published:** 2026-09-21

**Authors:** Norio Horiguchi, Etsuko Hisanaga, Hiroko Sato, Kuniko Yoshida, Akihito Kimura, Ayumi Ito, Miki Horita, Aya Suzuki, Keiko Kawai‐Kowase

**Affiliations:** ^1^ Department of General Medicine Gunma University Graduate School of Medicine Maebashi Japan; ^2^ Clinical Department of Pathology Gunma University Hospital Maebashi Japan

**Keywords:** arthrochalasia EDS, case report, Ehlers‐Danlos syndrome, joint instability, muscularis propria, small‐bowel perforation

## Abstract

**Background:**

Ehlers‐Danlos syndrome (EDS) may be overlooked when gastrointestinal and musculoskeletal findings are assessed separately.

**Case Presentation:**

An older woman with two previous small‐bowel perforations had bilateral congenital hip dislocation, recurrent shoulder dislocations, skin hyperextensibility, and muscularis propria thinning in nonperforated jejunum. Systematic reassessment using the 2017 classification met minimal clinical criteria suggestive of arthrochalasia EDS, while recurrent bowel perforation raised concern for vascular EDS. Genetic testing was declined.

**Conclusion:**

The overall phenotype supported clinically suspected EDS, but molecular confirmation was required for definitive subtype assignment.

## 1 Introduction

Ehlers‐Danlos syndrome (EDS) comprises 13 inherited connective‐tissue disorders characterized by variable skin, joint, and tissue‐fragility phenotypes [1]. Vascular EDS (vEDS) is associated with arterial and hollow‐organ rupture [1, 2], whereas arthrochalasia EDS (aEDS) is characterized by congenital bilateral hip dislocation, severe generalized joint hypermobility, multiple dislocations, and skin hyperextensibility [1]. Gastrointestinal perforation can prompt consideration of vEDS, although the 2017 major criterion specifically refers to unexplained sigmoid‐colon rupture [1]. We describe an older patient in whom recurrent small‐bowel perforations, lifelong joint instability, skin findings, and prior pathology were integrated, leading to clinically suspected EDS; systematic reassessment was clinically suggestive of aEDS, but no monogenic subtype could be confirmed.

## 2 Case Presentation

A woman in her 60s developed epigastric pain. Endoscopy showed a gastric ulcer, and esomeprazole was increased. Several days later, periumbilical and lower abdominal pain prompted emergency admission. Her temperature was 35.8°C, blood pressure 133/55 mmHg, pulse 76/min, and oxygen saturation 99% on room air. She had mild abdominal tenderness without rebound or guarding. Leukocyte count was 12.7 × 10^3^/μL and C‐reactive protein 11.2 mg/dL. Computed tomography showed perienteric fat stranding and mesenteric edema without free air or air‐fluid levels, suggesting enteritis. Symptoms improved with bowel rest, intravenous fluids, and antimicrobial therapy.

She had undergone surgery for small‐bowel perforation 15 years and 1 year earlier and had recurrent enteritis‐like episodes. Her history included bilateral congenital hip dislocation, left‐knee ligament surgery, right hip osteoarthritis surgery, recurrent bilateral shoulder dislocations requiring surgery, and lumbar spinal canal stenosis. Long‐term diclofenac use had previously raised concern for medication‐related intestinal injury. However, recurrent perforation and lifelong joint instability prompted reconsideration of a systemic connective‐tissue disorder.

**FIGURE 1:**
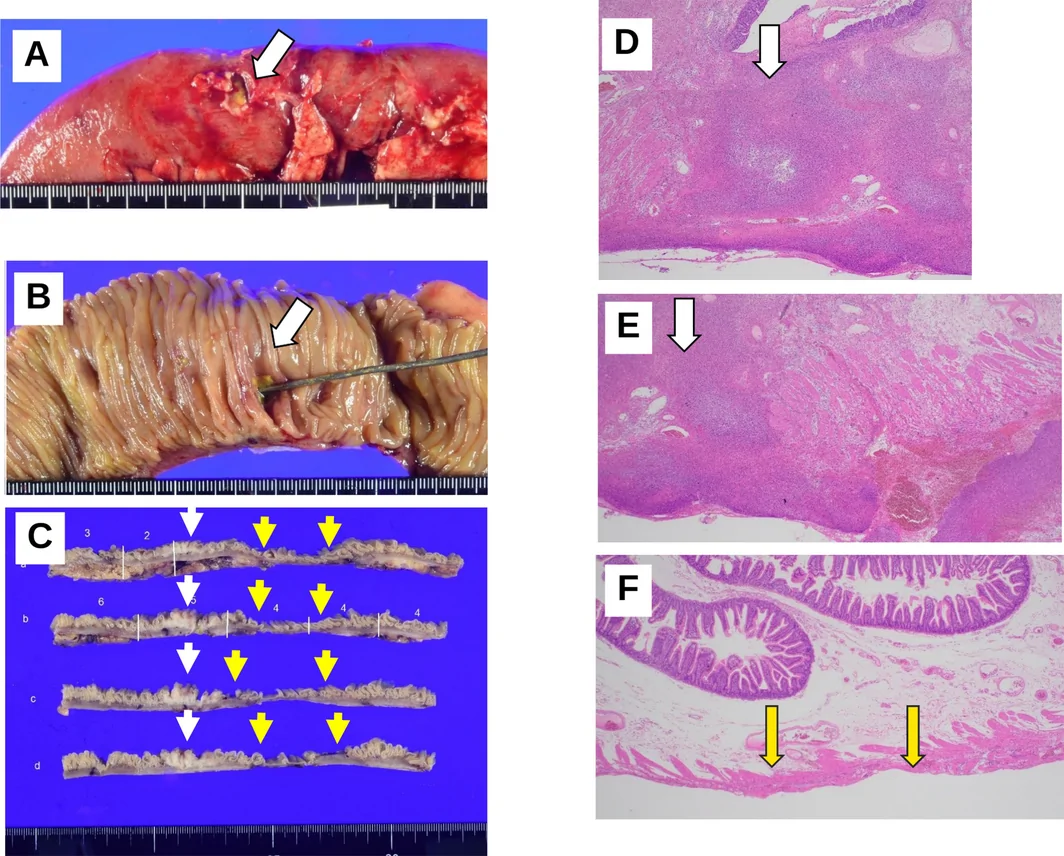
Resected jejunum. (A, B) Serosal and mucosal views of the perforation. (C) Serial sections showing perforation (white arrowheads) and wall thinning (yellow arrowheads). (D, E) Full‐thickness inflammation. (F) Markedly thinned muscularis propria in nonperforated proximal bowel (H&E).

Review of the prior jejunal resection showed a perforation with adjacent wall thinning (Figure 1A–C). The perforation site had full‐thickness inflammation, hemorrhage, exudate, and edema (Figure 1D,E). In nonperforated proximal bowel, the mucosa was relatively preserved while both layers of the muscularis propria were markedly thinned (Figure 1F). The original pathology report had documented this finding.

Dermatological assessment confirmed skin hyperextensibility. Finger joint hyperextension was absent at the current examination; historical Beighton scoring was unavailable. No easy bruising or overt skin fragility was noted, and cardiovascular and ophthalmologic evaluations showed no major abnormalities. Family history was unremarkable. The patient declined genetic testing after counseling. EDS was clinically suspected; the 2017 criteria were clinically suggestive of aEDS, but no monogenic EDS subtype could be confirmed without molecular testing.

## 3 Discussion

Systematic reassessment using the 2017 classification clarified both the support for EDS and the limits of subtype assignment (Table 1) [1]. For vEDS, bilateral congenital hip dislocation is a minor criterion, and recurrent intestinal perforation raised clinical concern; however, the two perforations involved small bowel rather than the sigmoid colon specified as a major criterion. She also had no recognized arterial event, easy bruising, thin translucent skin, or current small‐joint hypermobility. In contrast, bilateral congenital hip dislocation (major criterion 1) and skin hyperextensibility (major criterion 3) are both present for aEDS. According to the 2017 classification, this combination meets the minimal clinical criteria suggestive for aEDS. Recurrent bilateral shoulder dislocations provide additional phenotypic overlap, although severe generalized joint hypermobility was not formally established. Thus, aEDS is a clinically plausible subtype, whereas vEDS remains an important differential because of the recurrent bowel perforations. Molecular testing was declined, so neither monogenic subtype could be confirmed.

**TABLE 1:** Systematic clinical assessment according to the 2017 EDS classification.

Subtype	Relevant 2017 criteria	Findings in this patient	Interpretation
Vascular EDS (vEDS)	Major: (1) family history of vEDS with a documented causative COL3A1 variant; (2) arterial rupture at a young age; (3) spontaneous sigmoid‐colon perforation without known diverticular disease or other bowel pathology; (4) third‐trimester uterine rupture without previous cesarean section and/or severe peripartum perineal tears; (5) carotid‐cavernous sinus fistula without trauma. Minor: easy bruising; thin translucent skin; characteristic facial appearance; spontaneous pneumothorax; acrogeria; talipes equinovarus; congenital hip dislocation; small‐joint hypermobility; tendon/muscle rupture; keratoconus; gingival recession/fragility; early‐onset varicose veins.	Major features: family history unremarkable; no recognized arterial event; two recurrent small‐bowel perforations (not sigmoid); uterine rupture not applicable (no pregnancy history); carotid‐cavernous sinus fistula not documented. Minor features: bilateral congenital hip dislocation present; easy bruising absent; current small‐joint hypermobility absent; thin translucent skin and other characteristic minor features not documented.	Based on the available documentation, the 2017 minimal clinical criteria suggestive for vEDS are not met. Recurrent small‐bowel perforation remains clinically concerning for vEDS but is not the specific major criterion of unexplained sigmoid‐colon rupture. Molecular confirmation was not performed.
Arthrochalasia EDS (aEDS)	Major: (1) congenital bilateral hip dislocation; (2) severe generalized joint hypermobility with multiple dislocations/subluxations; (3) skin hyperextensibility. Minor: muscle hypotonia; kyphoscoliosis; radiologically mild osteopenia; tissue fragility including atrophic scars; easy bruising.	Major criterion 1: present (bilateral congenital hip dislocation). Major criterion 2: indeterminate; recurrent bilateral shoulder dislocations are documented, but severe generalized joint hypermobility was not formally assessed and historical Beighton scoring is unavailable. Major criterion 3: present (skin hyperextensibility). Minor criteria: easy bruising absent; hypotonia, kyphoscoliosis, mild osteopenia, and atrophic scarring/tissue fragility not documented.	The patient meets the 2017 minimal clinical criteria suggestive for aEDS through major criterion 1 plus major criterion 3. aEDS is therefore a clinically plausible subtype, but definitive diagnosis requires molecular confirmation of a causative COL1A1 or COL1A2 variant, which was not performed.
Classical EDS (cEDS)	Major: (1) skin hyperextensibility with atrophic scarring; (2) generalized joint hypermobility. Minor: easy bruising; soft/doughy skin; skin fragility; molluscoid pseudotumors; subcutaneous spheroids; hernia; epicanthal folds; complications of joint hypermobility; first‐degree family history meeting clinical criteria.	Skin hyperextensibility is present, but atrophic scarring is not documented; generalized joint hypermobility is not established. Recurrent dislocations are present as a complication of joint instability; easy bruising is absent; other minor features are not documented.	The required major criterion of skin hyperextensibility with atrophic scarring is not established; therefore, the 2017 minimal clinical criteria suggestive for cEDS are not met.
Hypermobile EDS (hEDS)	Criterion 1: generalized joint hypermobility. Criterion 2: at least two of Features A‐C (A, systemic manifestations; B, positive family history; C, qualifying musculoskeletal complications). Criterion 3: all prerequisites, including absence of unusual skin fragility and exclusion of alternative diagnoses.	Criterion 1: not established; current finger hyperextension is absent and historical Beighton score/5‐point questionnaire is unavailable. Criterion 2: recurrent bilateral shoulder dislocations are documented, but whether they were atraumatic is not documented; family history is unremarkable and sufficient systemic features for Feature A are not established. Criterion 3: cannot be fully assessed while alternative heritable connective‐tissue disorders remain under consideration.	The 2017 hEDS criteria cannot be fulfilled from the available records because generalized joint hypermobility is not established and the remaining criteria cannot be completely assessed.

*Note:* Criteria are summarized from Malfait et al. [1]. “Not documented” or “indeterminate” indicates that the available clinical record did not permit reliable assessment. For monogenic EDS subtypes, molecular confirmation is required for a definitive diagnosis.

Abbreviations: aEDS, arthrochalasia Ehlers‐Danlos syndrome; cEDS, classical Ehlers‐Danlos syndrome; EDS, Ehlers‐Danlos syndrome; hEDS, hypermobile Ehlers‐Danlos syndrome; vEDS, vascular Ehlers‐Danlos syndrome.

The absence of recognized arterial complications into her 60s is less typical of classic vEDS but does not exclude the molecularly confirmed spectrum. In 1231 individuals with COL3A1 variants, disease severity and survival varied substantially by variant type [3]. In a recent Dutch cohort of 142 genetically confirmed patients, the median age at first major event was 41 years, but later events occurred, particularly with presumed haploinsufficient variants, for which the median was 53 years [4]. These data support phenotypic variability while underscoring that late presentation cannot be used to infer vEDS without molecular confirmation.

The pathological findings also require restraint. Muscularis‐propria thinning or segmental absence of intestinal musculature has been reported in vEDS‐associated perforation [5, 6], but it is not diagnostic and may reflect inflammation, ischemia, medication‐related injury, or a primary muscular defect. Chronic NSAID exposure was a plausible contributor in this patient. Nevertheless, marked muscular thinning in nonperforated bowel, interpreted together with the multisystem phenotype, supported the possibility of underlying bowel‐wall fragility rather than localized injury alone.

For generalists, the principal lesson is longitudinal integration: recurrent gastrointestinal perforation, joint instability, skin findings, and older pathological information may collectively reveal an inherited connective‐tissue disorder even when no single finding is diagnostic.

## 4 Conclusion

Recurrent unexplained gastrointestinal perforations with long‐standing joint instability and skin hyperextensibility should prompt consideration of EDS, even in older adults. In this patient, systematic assessment met the minimal clinical criteria suggestive for aEDS, while recurrent bowel perforation also raised concern for vEDS. Because molecular testing was declined, the specific monogenic EDS subtype could not be confirmed.

## Author Contributions

**Norio Horiguchi:** conceptualization, methodology, data curation, investigation, visualization, writing – original draft, writing – review and editing. **Etsuko Hisanaga:** methodology, data curation, investigation, writing – review and editing. **Hiroko Sato:** data curation, investigation, writing – review and editing. **Kuniko Yoshida:** data curation, writing – review and editing, investigation. **Akihito Kimura:** investigation, writing – review and editing, data curation. **Ayumi Ito:** investigation, writing – review and editing, data curation. **Miki Horita:** investigation, writing – review and editing, data curation. **Aya Suzuki:** methodology, investigation, data curation, writing – review and editing. **Keiko Kawai‐Kowase:** conceptualization, methodology, data curation, visualization, writing – review and editing, investigation, supervision.

## Funding

The authors have nothing to report.

## Ethics Statement

Written informed consent was obtained from the patient for publication of this case report and the accompanying images.

## Conflicts of Interest

The authors declare no conflicts of interest.

## Data Availability

Data sharing not applicable to this article as no datasets were generated or analysed during the current study.
